# Long-term biochemical progression-free survival following brachytherapy for prostate cancer: Further insight into the role of short-term androgen deprivation and intermediate risk group subclassification

**DOI:** 10.1371/journal.pone.0215582

**Published:** 2019-04-19

**Authors:** Haim Matzkin, Juza Chen, Rubi Agai, Tomer Ziv-Baran, Nicola J. Mabjeesh

**Affiliations:** 1 Department of Urology, Tel Aviv Sourasky Medical Center, Sackler Faculty of Medicine, Tel Aviv University, Tel Aviv, Israel; 2 The Institute of Radiotherapy, Department of Oncology, Tel Aviv Sourasky Medical Center, Tel Aviv, Israel; 3 School of Public Health, Sackler Faculty of Medicine, Tel Aviv University, Tel Aviv, Israel; Barretos Cancer Hospital, BRAZIL

## Abstract

**Introduction:**

Brachytherapy is a well-established treatment of localized prostate cancer. Few studies have documented long-term results, specifically biochemical progression-free survival (bPFS) in men with brachytherapy alone, with or without short-term androgen deprivation therapy (ADT), or in combination with external beam radiotherapy (EBRT). Our aim was to analyze long-term bPFS of brachytherapy treated patients.

**Materials and methods:**

Retrospective analysis of 1457 patients with low and intermediate risk prostate cancer treated with brachytherapy alone (1255) or combined with EBRT (202). Six-months ADT was administrated for all EBRT combined patients and for prostate volume downsizing when >55 cc (328). Failure was by the Phoenix definition. Kaplan-Meier analysis and multivariate Cox regression estimated and compared 10-yr and 15-yr rates of bPFS.

**Results:**

Median follow-up was 6.1 yr. Ten and 15-yr bPFS rates of the entire cohort were 93.2% and 89.2%, respectively. On multivariate analysis, PSA density (PSAD), ADT and clinical stage were significantly associated with failure. The most powerful independent factor was PSAD with a HR of 3.5 (95% CI, 1.7–7.4) for PSAD above 0.15. No significant difference was found between low and intermediate risks patients regardless of treatment regimen. However, comparison of two intermediate risk groups, Gleason score (GS) 7, PSA<20 ng/ml versus GS≤6 and PSA = 10–20 ng/ml, revealed 10- and 15-yr bPFS rates of 94.2% and 94.2% compared to 88.2% and 79.9%, (*P* = 0.022), respectively. ADT improved bPFS rates in low risk patients. The ten and 15-yr bPFS rates were 97.6% and 94.6% compared to 92.3% and 88.2%, (*P* = 0.020), respectively.

**Conclusions:**

Our retrospective large scale study suggests that brachytherapy provides excellent long-term bPFS rates in low and intermediate risk disease. Combination of brachytherapy with EBRT yields favorable outcomes in GS 7 intermediate risk patients and short-term ADT has a positive effect on outcomes in low risk patients.

Further prospective studies are warranted to discriminate the role of adding either EBRT and/or ADT to brachytherapy protocols.

## Introduction

Since 1983 a large number of patients have been treated with brachytherapy, generating a large database in many excellence centers [1]. However no randomized controlled studies have been published comparing long-term oncological results of brachytherapy to other current treatment approaches. Therefore high volume and longer prospective follow up reports are welcome to substantiate this treatment modality and perhaps answer some still debated issues, such as the role of adjuvant short term androgen deprivation therapy (ADT), and of combined external beam radiotherapy (EBRT) in appropriate patients. The RTOG 0232 randomized trial aiming to answer the clinical dilemma of treating Gleason score (GS) 7 by brachytherapy or brachytherapy and EBRT has recently been published as Abstract only [2]. Many brachytherapy centers have moved towards the use of brachytherapy only, in cases with intermediate D'Amico risk group patients and do not reserve brachy-monotherapy for low risk patients only [3]. This approach is backed-up by the excellent clinical results achieved with monotherapy in low risk patients, the ever improving computation capabilities in the operating room and the trend for lower risk patients being diagnosed as a result of the widespread use of PSA.

We had a unique opportunity to try and clarify some of the still open issues, not only having treated and prospectively followed-up a rather large cohort of patients but because we segregated our intermediate risk group into two subgroups (dissimilar to the D'Amico grouping): those with Gleason score 6, PSA 10–20 ng/ml being treated with brachytherapy only while all Gleason score 7 patients were treated with brachytherapy and EBRT. Additionally, we tried to elucidate the role of short-term ADT in the low risk patients treated with brachytherapy only.

## Materials and methods

### Patient population and classification of biochemical failure

The institutional review board approved this study and waived informed consent requirements. A total of 1552 consecutive patients with biopsy-proven prostate cancer were treated with transperineal ^125^I-based permanent brachytherapy between 2000 and 2016. Men less of eighty years with Gleason score ≤ 6, clinical stage T_1_–T_2_ and PSA < 20 ng/ml were given ^125^I brachytherapy as monotherapy. Men with Gleason score 7, of the same stage or PSA values were given a combination of ^125^I brachytherapy (100% isodose of 96–107 Gy) and EBRT, 45 Gy at a daily dose of 1.8 Gy. ADT was given either to reduce gland size, if gland volume was above 55 cc, or to all men with Gleason 7 disease treated with combined EBRT, as adjuvant treatment. ADT consisted of two 3 monthly GNRH-A injections and oral bicalutamide given for the first month only. Patients were treated using the real-time intraoperative methodology [4–6]. Patient selection criteria and risk stratification for monotherapy vs. combined treatment were established at the start of our program and remained constant. Patients were seen at 1, 3, 6, 9, 12 18, 24 months and every 6–12 months thereafter. CT dosimetry, was obtained at 1 month. Biological effective dose (BED) calculations were performed using equations previously described [7,8]. Serum PSA was determined at each visit from month 3 onwards. Biochemical failure was defined using the Phoenix definition of a rise by 2 ng/mL or more above the nadir PSA [9]. Out of 1552 patients, only 95 (6%) were excluded from the study because their data after treatment were not available for analysis due to lost to follow-up from any reason.

### Brachytherapy method

The intraoperative methodology described in detail by Stone, Stock and coworkers [4–6] bases its dosimetry calculations in the operating room after the patient has been positioned and needles inserted. It relies heavily on rapid software and physicist calculations, and optimal dosimetry is decided only then and executed in real-time. Identical B&K ultrasound unit (Bruel & Kjaer, Gentofte, Denmark) was used for all patients as well as dedicated software (Varian Medical Systems, Inc., Palo Alto, CA). All patients received the same postoperative care (a minimum of 1 month α-blockers as default) and follow-up.

### Statistical analyses

Categorical variables were expressed as number and percentages. Distribution of continuous variables was assessed using histogram and Q-Q plot. Normally distributed continuous variables were described using mean and standard deviation (SD), and non-normally distributed continuous variables were expressed using median and interquartile range (IQR). We assessed differences in the baseline characteristics between the patients included in the study and those who were excluded, using Chi-square test or Fisher’s exact test for categorical variables and independent samples t-test or Mann-Whitney test for continuous variables, as appropriate. A Kaplan–Meier plot was used to describe the biochemical progression-free survival (bPFS) between categories and the log-rank test was applied to compare between them. Follow up time was obtained using reverse censoring method. Univariate and multivariate Cox regression were used to evaluate the association between mortality and potential predictors. Backward stepwise (Wald) method was used for variables selection. Variables with clinical significance and with *P* value < 0.1 on univariate analysis were a priori selected for inclusion in the multivariate analysis; first and last steps are presented. The plot of the Schoenfeld residuals versus time and the log minus log plot were used to check for the proportional hazard assumption. In addition, the Schoenfeld residuals were regressed against time to test for independence between residuals and time. A two-tailed *P* < 0.05 was considered statistically significant. Analyses were performed with SPSS (IBM Corp. Released 2013. IBM SPSS Statistics for Windows, Version 22.0. Armonk, NY: IBM Corp.).

## Results

### Patient and primary treatment characteristics

Patient allocation are summarized in a CONSORT (Consolidated Standards Of Reporting Trials) diagram (S1 Fig). Patient and treatment characteristics are presented in Tables 1 and 2, respectively. Median follow-up of the overall cohort was 6.08 yr (IQR, 3.16–9.08 yr). Most patients (68%) had low risk prostate cancer according to D’Amico stratification system; 83.6% had Gleason score 6 or less, 84.6% had initial PSA less than or equal to 10 ng/ml and 94% clinical stage T1c and T2a. Two hundred and two out of 239 patients (84.5%) with Gleason score 7 were treated with brachytherapy combined with EBRT and 6 months ADT. The remaining 37 men were either contraindicated to receive EBRT due to previous pelvic irradiation or colonic surgery or declined the combination suggested. ADT was also given to patients whose prostate volume was more than 55 cc at the initial TRUS measurement. One month post brachytherapy dosimetry analysis revealed that most of the patients received optimal amounts of radiation with a median BED of 241 Gy (Table 2).

**Table 1:** Clinical and pathologic characteristics (n = 1457).

Age, yr, mean (SD)		66.7 (6.5)
PSA, ng/ml, median (IQR)		6.53 (5.10-8.72)
Gleason score, n (%)		
	≤ 6	1218 (83.6)
	7	239 (16.4)
Clinical stage, n (%)		
	T1c	1102 (75.6)
	T2a	267 (18.4)
	T2b	56 (3.8)
	Tx	32 (2.2)
Risk group, n (%)		
	Low	991 (68.0)
	Intermediate	441 (30.3)
	NA	25 (1.7)
PPC, median (IQR)		25 (14.3-40.0)
Prostate volume at treatment, ml, mean (SD)		38.1 (9.4)
PSAD, ng, mean (SD)		0.20 (0.11)
EBRT, n, (%)		202 (13.9)
ADT, n, (%)		531 (36.4)

SD = standard deviation; IQR = interquartile range; PSA = prostate-specific antigen; EBRT = external beam radiation therapy; ADT = androgen deprivation therapy; NA = not applicable; PSAD = PSA density; PPC = percent of positive cores.

**Table 2:** Treatment details (n = 1457).

Seed number, mean (SD)	66.8 (12.7)
Seed specific activity, mCi, mean (SD)	0.456 (0.058)
Postimplant prostate volume, ml, mean (SD)	41.7 (11.4)
V100, (%), median (IQR)	96.70 (95.51-98.00)
V90, (%), median (IQR)	98.80 (98.09-99.40)
BED (Gy), median (IQR)	241 (229-269)
D90 (Gy), median (IQR)	
No EBRT	182.62 (175.94-191.19)
Combined with EBRT	126.69 (121.21-134.44)

SD = standard deviation; IQR = interquartile range; EBRT = external beam radiation therapy; V100 = Volume of the prostate receiving 100% of the prescription dose; V90 = volume of the prostate receiving 90% of the prescription dose; D90 = radiation dose delivered to 90% of the prostate; BED = biological effective dose.

### Univariate and multivariate analyses of biochemical progression-free survival

During follow-up 55 patients failed biochemically according to the Phoenix definition, two of them developed metastatic disease and 172 patients died, none of prostate cancer. On univariate analysis, PSA, prostate volume at treatment, PSA density (PSAD) and clinical stage were significantly associated with biochemical failure (Table 3). ADT and percent of positive cores (PPC) were nearly significant predictive factors. ADT had a protective effect from failure whereas PPC > 50 increased the risk for failure (Table 3). Of note, EBRT had no impact on bPFS. On Cox multivariate regression analysis the following factors were included: age, PSA, Gleason score, PSAD, disease stage, BED, ADT and PPC. PSAD and clinical stage remained significantly associated with failure on multivariate analysis whereas ADT was with tendency to significance (Table 4). The most powerful independent factor was PSAD with a HR of 3.463 (95% CI, 1.628–7.364) for patients with PSAD > 0.15.

**Table 3:** Univariate Cox regression of biochemical progression-free survival.

Parameter	HR (95% CI)	*P* value
Age	1.010 (0.968 -1.053)	0.654
PSA	1.085 (1.008- 1.168)	0.029
Prostate volume at treatment	0.954 (0.929-0.980)	0.001
V100	0.980 (0.900- 1.067)	0.64
V90	1.018 (0.862-1.203)	0.832
BED	1.005 (0.995-1.014)	0.337
Gleason score	1.020 (0.626-1.662)	0.936
EBRT	0.678 (0.270-1.699)	0.407
ADT	0.607 (0.339-1.087)	0.093
PSAD (> 0.15 vs. ≤ 0.15)	3.490 (1.648-7.390)	0.001
Clinical stage		0.035
T1c (Ref.)		
T2a	2.048 (1.161-3.615)	0.013
T2b	1.959 (1.692-5.545)	0.205
Risk group (intermediate vs. low)	1.486 (0.857-2.576)	0.158
PPC (≤ 50 vs. > 50)	1.801 (0.906-3.579)	0.093

HR = hazard ratio; CI = confidence interval; PSA = prostate-specific antigen; EBRT = external beam radiation therapy; ADT = androgen deprivation therapy; V100 = Volume of the prostate receiving 100% of the prescription dose; V90 = volume of the prostate receiving 90% of the prescription dose; D90 = radiation dose delivered to 90% of the prostate; BED = biological effective dose; PSAD = PSA density; PPC = percent of positive cores.

**Table 4:** Multivariate Cox regression of biochemical progression-free survival.

Step	Parameter	HR (95% CI)	*P* value
First step			
	Age	1.000 (0.957-1.045)	0.997
	PSA	1.043 (0.941-1.157)	0.422
	PSAD (> 0.15 vs. ≤ 0.15)	2.939 (1.255-6.882)	0.013
	PPC (≤ 50 vs. > 50)	1.439 (0.696-2.973)	0.326
	Gleason score (7 vs. ≤ 6)	0.523 (0.165-1.657)	0.271
	ADT	0.618 (0.308-1.239)	0.175
	BED	1.003 (0.993-1.012)	0.562
	Clinical stage		0.033
	T1c (Ref.)		
	T2a	2.078 (1.149-3.759)	0.016
	T2b	2.337 (0.773-7.065)	0.132
Last step			
	PSAD (> 0.15 vs. ≤ 0.15)	3.463 (1.628-7.364)	0.001
	Gleason score (7 vs. ≤ 6)	0.375 (0.135-1.061)	0.065
	Clinical stage		0.016
	T1c (Ref.)		
	T2a	2.176 (1.212-3.907)	0.009
	T2b	2.621 (0.908-7.567)	0.075

HR = hazard ratio; CI = confidence interval; PSA = prostate-specific antigen; PSAD = PSA density; ADT = androgen deprivation therapy; BED = biological effective dose; PPC = percent of positive cores.

### Biochemical progression-free survival outcome

**Fig 1:**
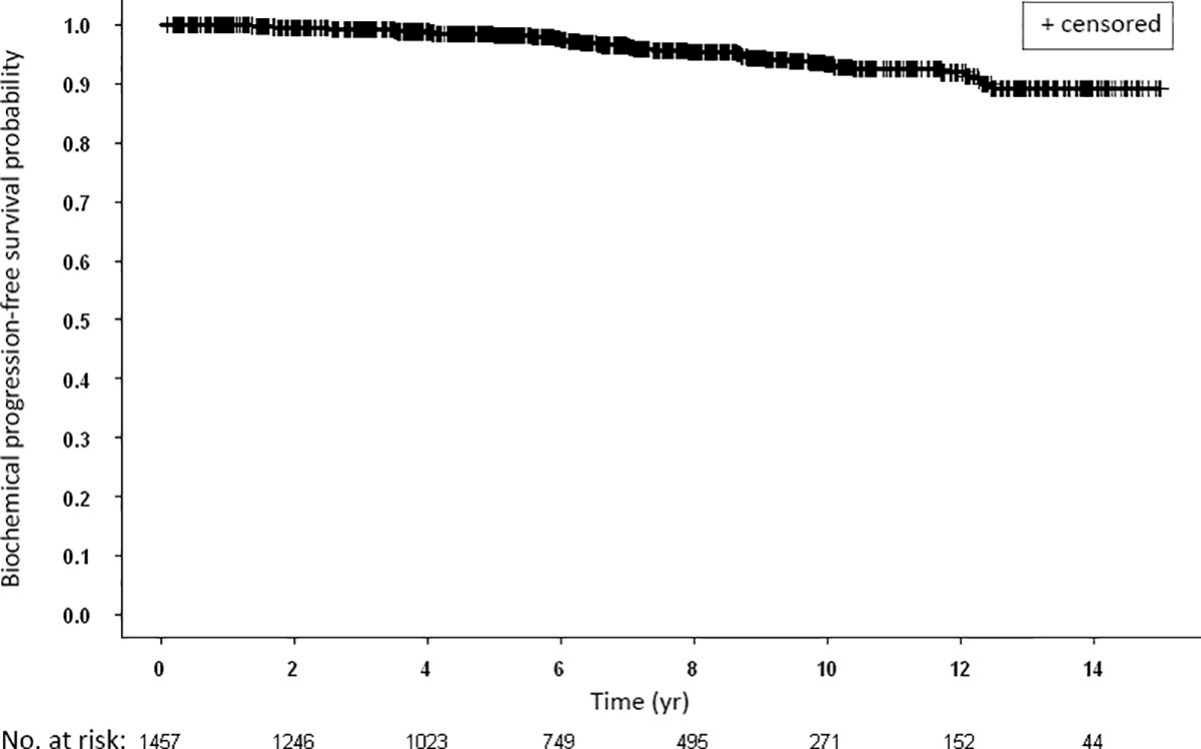
Kaplan-Meier curves for biochemical progression-free survival of all study patients.

Ten and 15-yr bPFS rates of the entire cohort were 93.2% and 89.2%, respectively, as shown by Kaplan-Meier analyses (Fig 1). In addition, we analyzed PSA nadir values as a predictor for disease-free survival and found that 88.1% of patients had a nadir value < 0.5 ng/ml.

### Prognostic significance of PSA

**Fig 2:**
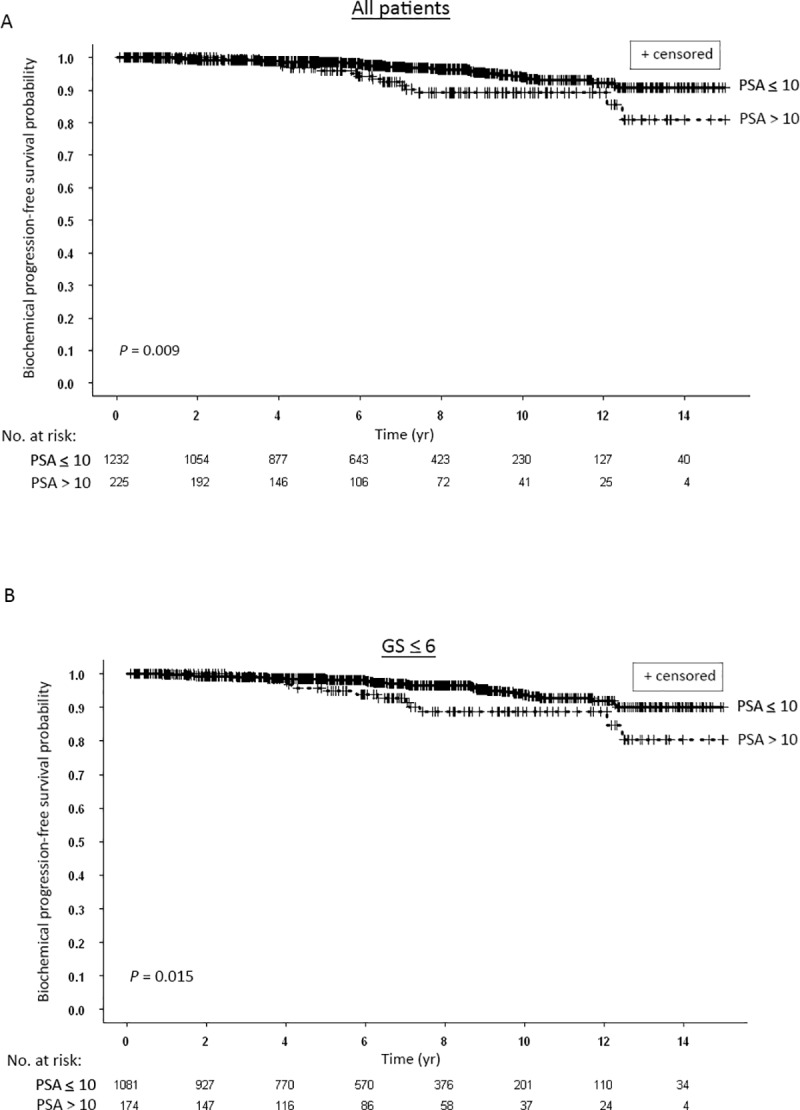
(A) Kaplan-Meier curves for biochemical progression-free survival of all patients stratified according to pretreatment blood prostate-specific antigen (PSA) levels: ≤ 10 ng/ml vs. > 10 ng.ml. (B) Kaplan-Meier curves for biochemical progression-free survival of patients with Gleason score (GS) ≤ 6 stratified according to pretreatment blood PSA levels: ≤ 10 ng/ml vs. > 10 ng.ml.

There were significant differences between the Kaplan-Meier bPFS rates for patients with initial PSA values > 10 ng/ml compared to patients with PSA ≤ 10 ng/ml (Fig 2A). Ten and 15-yr bPFS rates were 89.2% and 81.0% compared to 93.9% and 90.7%, respectively (Fig 2A). Similar significant difference was also seen in the subgroup of patients with Gleason score ≤ 6 (Fig 2B).

### Prognostic significance of Gleason score and risk stratification

**Fig 3:**
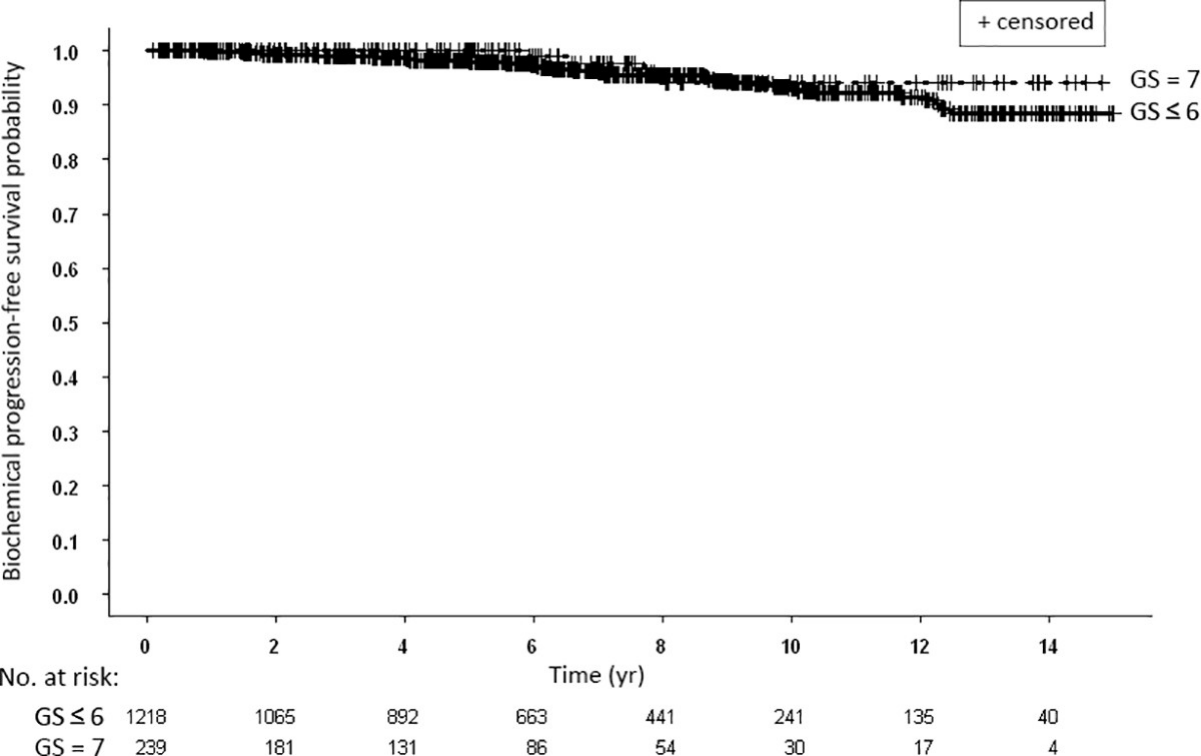
Kaplan-Meier curves for biochemical progression-free survival of all patients stratified according to Gleason score (GS): ≤ 6 vs. 7.

**Fig 4:**
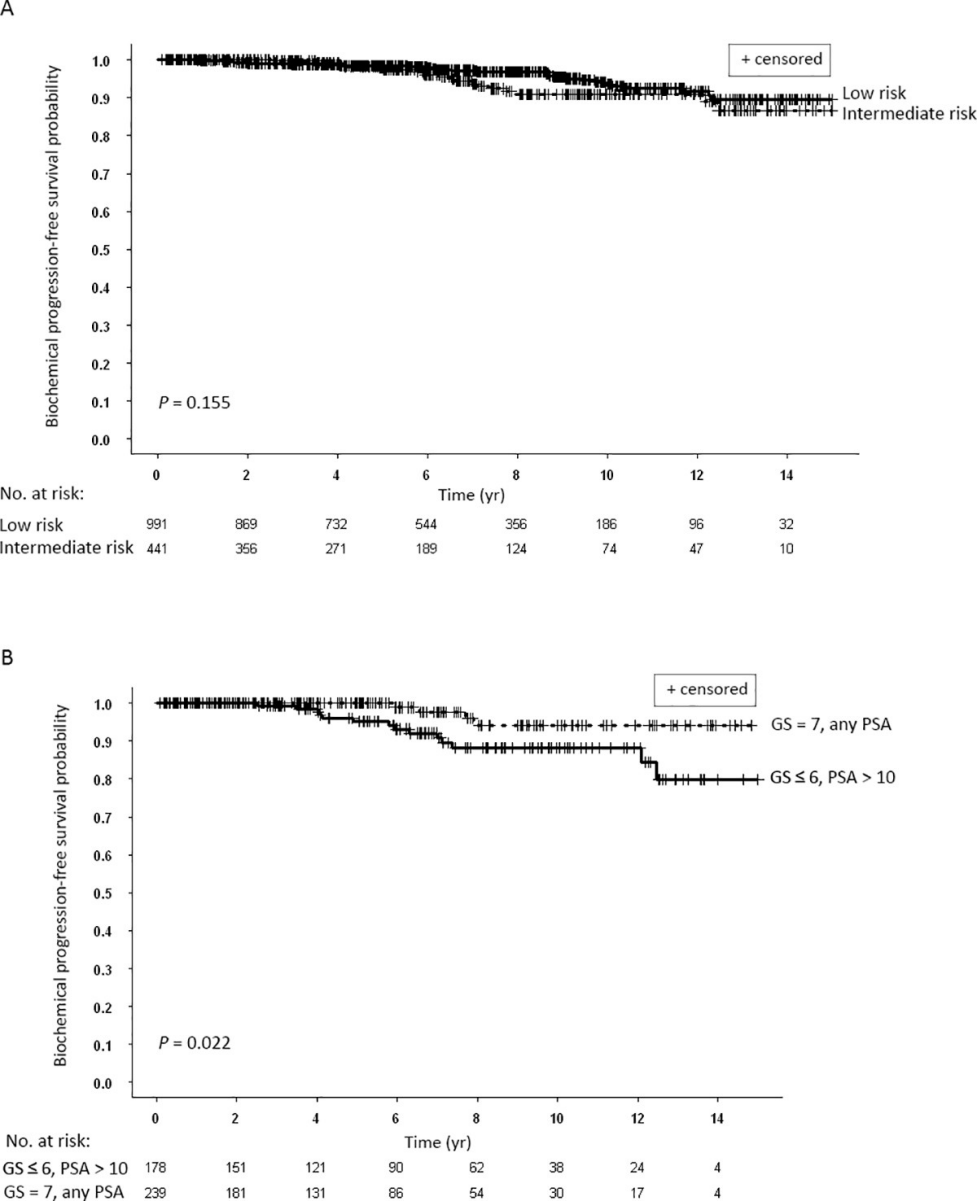
(A) Kaplan-Meier curves for biochemical progression-free survival of all patients stratified according to D’Amico risk grouping: low vs. intermediate. (B) Kaplan-Meier curves for biochemical progression-free survival of two intermediate risk groups: patient with Gleason score (GS) ≤ 6 and pretreatment blood prostate-specific (PSA) levels > 10 ng.ml vs. patients with GS = 7 and any pretreatment PSA levels.

Patients with Gleason score 7 treated with combination radiotherapy and patients with Gleason score ≤ 6 treated with brachytherapy only had similar bFFS (Fig 3). Ten and 15-yr bPFS rates were 94.2% and 94.2% and 93.0% and 88.5%, respectively. When patients were divided according to their disease risk, there was no significant difference in the bPFS rates between low and intermediate risk groups (Fig 4A). Comparison of the two subpopulations of the intermediate risk group, patients with Gleason score 7, any PSA or stage versus patients with Gleason score ≤ 6 and PSA > 10 ng/ml, treated a priori differently, yielded significantly different bPFS rates (Fig 4B). Ten and 15-yr bPFS rates were 94.2% and 94.2% compared to 88.2% and 79.9%, respectively.

### Prognostic significance of PSA Density (PSAD)

**Fig 5:**
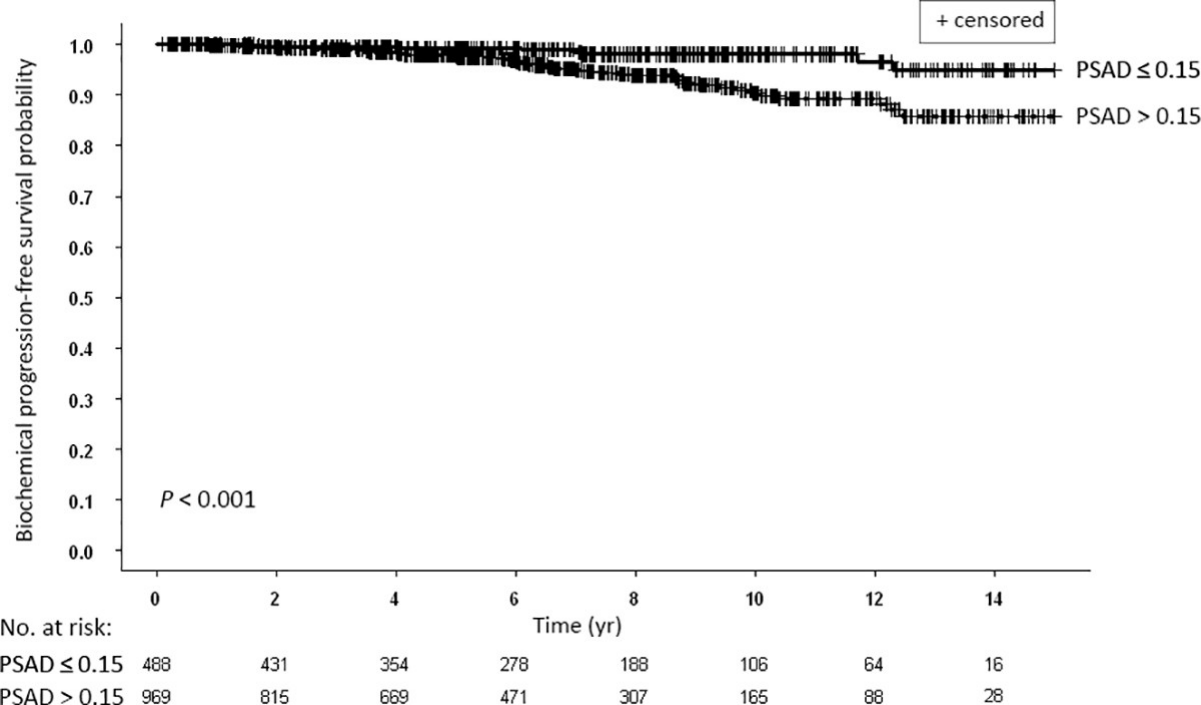
Kaplan-Meier curves for biochemical progression-free survival of all patients stratified according to prostate-specific antigen density (PSAD): ≤ 0.15 vs. > 0.15.

We next examined the significance of PSAD on biochemical recurrence. There were significant differences between the Kaplan-Meier bPFS rates for patients with PSAD > 0.15 ng compared to patients with PSAD ≤ 0.15 ng (Fig 5). The ten and 15-yr bPFS rates were 90.4% and 85.8% compared to 98.1% and 94.9%, respectively.

### Prognostic significance of ADT

**Fig 6:**
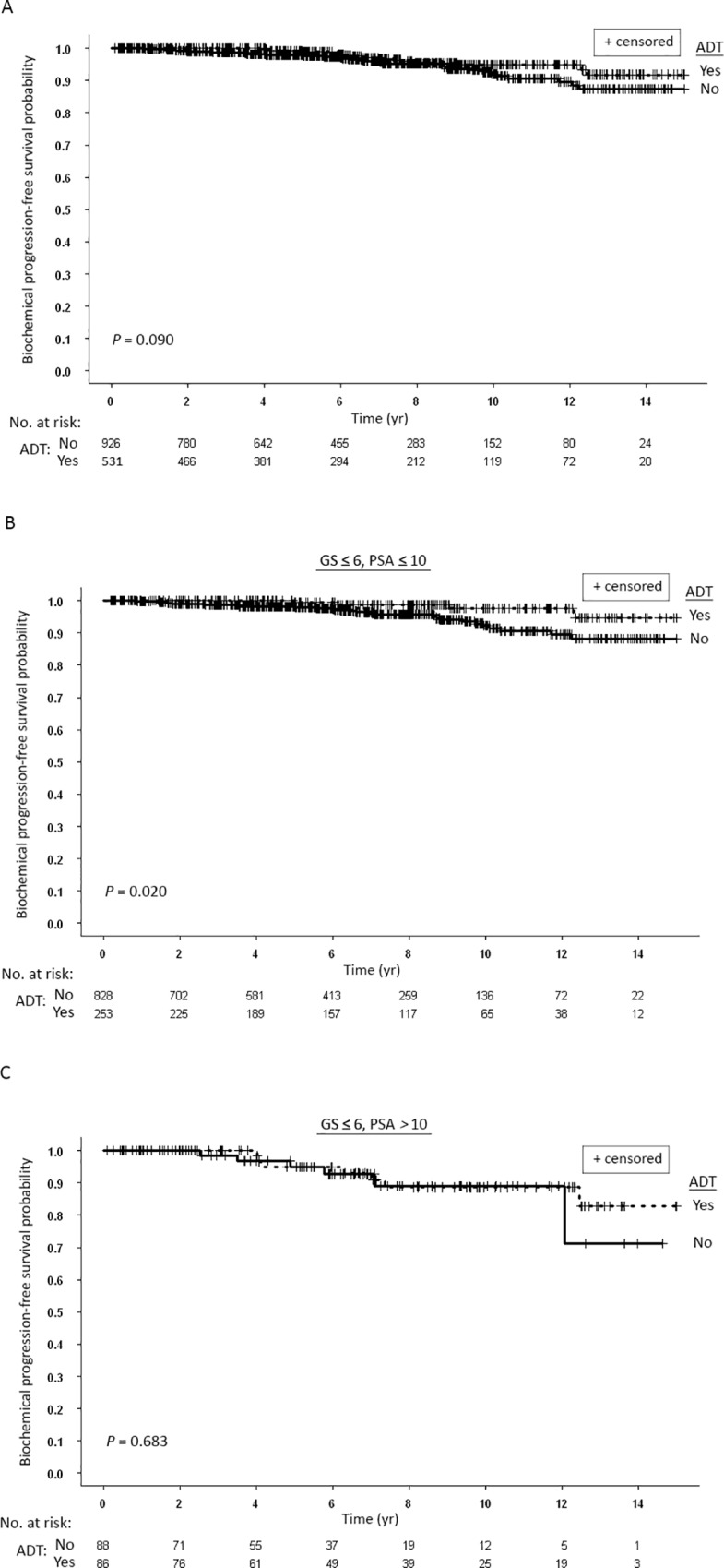
(A) Kaplan-Meier curves for biochemical progression-free survival of all patients stratified according to whether they received (Yes) androgen deprivation therapy (ADT) or did not receive ADT (No). (B) Kaplan-Meier curves for biochemical progression-free survival of patients with Gleason score (GS) ≤ 6 and prostate-specific antigen (PSA) ≤ 10 ng/ml stratified according to whether they received (Yes) ADT or did not receive ADT (No). (C) Kaplan-Meier curves for biochemical progression-free survival of patients with Gleason score (GS) ≤ 6 and PSA > 10 ng/ml stratified according to whether they received (Yes) ADT or did not receive ADT (No).

In our population, there were nearly significant differences between the bPFS rates for patients who received ADT compared to patients who did not (Fig 6A). The ten and 15-yr bPFS rates were 94.9% and 91.8% compared to 92.1% and 87.3%, respectively. Of note, ADT among patients with Gleason ≤ 6 was administered based only on the size of the gland. Among the whole group of Gleason ≤ 6 patients, there was no influence of ADT on bPFS (not shown). However, the differences in bPFS were statistically significant in the low risk subgroup of patients with Gleason score ≤ 6 and PSA ≤ 10 ng/ml (Fig 6B) compared to intermediate risk subgroup of patients Gleason score ≤ 6 and PSA > 10 ng/ml (Fig 6C).

### Prognostic significance of PPC

**Fig 7:**
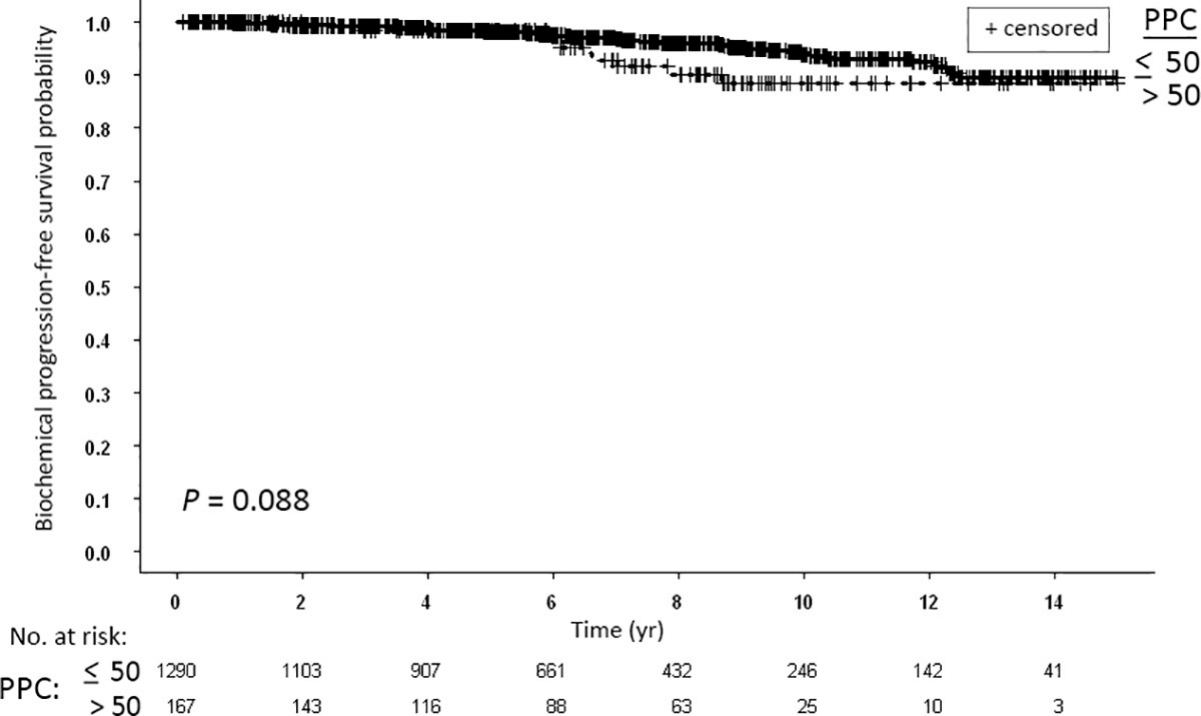
Kaplan-Meier curves for biochemical progression-free survival of all patients stratified according to the prostate biopsy percent of positive cores (PPC): ≤ 50 vs. > 50.

In our cohort, the effect of PPC on patients’ outcome was weak (Fig 7). There were nearly significant differences between the bPFS rates for patients with PPC > 50 compared to patients with PPC ≤ 50 (Fig 7). The ten and 15-yr bPFS rates were 88.4% and 88.4% compared to 93.9% and 89.6%, respectively.

## Discussion

Prostate brachytherapy for low to intermediate risk cancer, has been demonstrated by many to be as effective as EBRT and as good as surgery [10]. Brachytherapy alone has traditionally been selected for low risk prostate cancer and more recently for intermediate risk patients too [11,12], although many authorities still advocate the use of combined brachytherapy and EBRT +/- short term ADT for intermediate and high risk disease [13].

While it is certainly desirable, evidence for the relative merits and demerits of brachytherapy based on randomized trials is almost non-existent and Giberti's et al. recent publication on a small group of 165 randomly assigned men to either surgery or brachytherapy showing similar bPFS is a welcome addition [14]. We are therefore left with large series emanating from excellence centers with 7–12 yr follow-up reports, as the sole basis for the evaluation of the role of brachytherapy in the different prostate cancer risk categories [15–17]. Our series, more contemporary, perhaps the only one in the “active surveillance era” further selecting the lower risk population, demonstrates similar oncological outcomes to the published ones, with a bPFS of 98.1%, 93.2% and 89.2% at 5, 10 and 15 years, respectively. Our excellent overall bPFS attest probably not only to the local expertise, but also to careful case selection and the fact that in this more contemporary cohort than the ones previously published, the worldwide trend of lower and better risk, lower volume disease might have positively affected our presented outcomes.

Grimm et al. reported an overview showing that LDR (or HDR) is equivalent to combined LDR brachytherapy with EBRT for intermediate risk disease [18]. Indeed, many often use brachytherapy as monotherapy in selected favorable intermediate cases. Unfortunately, all reports are retrospective and include in their intermediate risk group both Gleason score 7 disease and Gleason score 6 PSA 10–20 ng/ml patients [19,20].

The D'Amico intermediate-risk group, as currently defined is a rather heterogeneous one. We initially believed that any men with Gleason score 7 should be treated differently and more aggressively than men with Gleason 6 and a PSA 10–20 ng/ml. Therefore men with Gleason score 6 and a PSA 10–20 ng/ml (D’Amico intermediate risk group) were given brachytherapy as monotherapy while all men with Gleason score 7 disease PSA < 20 ng/ml (also D’Amico intermediate risk group) were treated by combined radiation. Our subclassification is analogous to similar attempts published in recent years [21,22]. However, our definition as well as the others, for more favorable intermediate risk groups has not been validated and certainly needs better clarification. The current segregation into Gleason score 3+4 vs. 4+3 certainly does not apply to our group since at the time, this pathological difference was still not practiced.

Amini et al. have recently analyzed data from the National Cancer Database [23]. Out of a total of 10,571 intermediate risk group patients, 3,148 received combination treatment and 7,423 brachytherapy only. With a median follow up of 7 years they describe a modest overall survival benefit for the combination arm. In a subset analysis based on the Radiation Therapy Oncology Group (RTOG) 0232 trial inclusion criteria, much in accordance with our practice (Gleason score 7 vs. Gleason score < 7 and PSA 10–20 ng/ml) they demonstrated similar overall survival with the combination therapy and question the clinical importance of the small survival benefit faced with potential more side effects. The RTOG 0232 randomized trial results presented recently in ASTRO 2018 suggest similar segregation into the group of intermediate risk patients show that EBRT combined with brachytherapy is not necessary in favorable intermediate group patients [2] while ASCENDE-RT trial showed improved bPFS with brachytherapy boost given with EBRT + ADT compared to EBRT + ADT alone for intermediate and high risk disease [24].

Our results substantiate the need to treat intermediate risk patients, as currently segregated with combined radiotherapy, as those with Gleason score 6 with a PSA between 10 and 20 ng/ml treated with brachytherapy monotherapy fared less well than those belonging to the intermediate risk group based on the Gleason score 7 disease and treated with combination of EBRT (15-year bPFS was 79.9% vs. 94.2%, *P* = 0.022, respectively). This statistically significant difference was observed in spite of excellent D90/BED CT based dosimetry obtained in the brachytherapy only group. Our long-term data, albeit retrospective, support the need for combination therapy to achieve the best clinical outcomes. As a caveat mpMRI evaluating volume of disease and local extent of disease was not in use in our cohort started in year 2000 and therefore one cannot rule out that some of our Gleason score 6 patients with a higher than 10 ng/ml PSA, had a higher volume of disease perhaps with capsular involvement in which the addition of EBRT was of critical importance. This is certainly substantiated by our observation on the prognostic significance of PSAD (a proxy of disease volume). There is always the issue of the risk of the primary tumor being underestimated by the randomly taken biopsies, especially as PSA and disease volume is higher. This perhaps leads to undertreatment in many cases that may be compensated by the combination therapy.

The addition of 6- or more months ADT to EBRT is now well-established for intermediate to high risk localized prostate cancer with demonstrated improved overall survival [25]. The recent recognition of ADT negative side-effects, we believe justifies searching for clear cut evidence favoring addition of ADT to brachytherapy in low-risk patients [26].The added value of short-term ADT in low risk disease and among brachytherapy treated men has not been proven. Some argue that the extremely high BED achieved with the current real-time implant methodology negates the need of hormonal treatment even in intermediate and high risk men [3,27]. Others, that hormonal treatment is still helpful regardless of the BED achieved since it puts the cancer cells in the vulnerable cell cycle status, much in accordance with what is currently accepted for EBRT [27,28]. By contrast, Beyer et al. even reported worse overall survival in men receiving neoadjuvant ADT before brachytherapy [29]. Dickinson et al. [30] reported on a thousand low-risk patients among them 22% received short term ADT for gland downsizing baseline. In a rather short-term follow up of less than 5 years they found on a multivariate analysis that the absence of ADT was predictive for failure (96.8% vs. 93.4% bPFS, *P* = 0.033). The divergence between the Kaplan-Meier curves relating to ADT treatment becomes prominent after 4–5 years. The length of ADT was not reported by the authors and may be crucial especially in the short-term follow up period. The fact that their post implant CT based dosimetry D90 was only 124.9 Gy may further explain the positive role of ADT in their low risk patients, while we as well as other report much higher D90 outcomes which may negate the need for auxiliary ADT. It is important to emphasize that a recent systemic review from the American Brachytherapy Society Task Group Report analyzing data of more than 40,000 patients concluded that there is no benefit in adding ADT to prostate brachytherapy in low and favorable intermediate risk patients [31].

Among our low-risk men treated by brachy-montherapy, the addition of a 6-month ADT course given for cytoreductive purposes did make a difference seen in the long term in bPFS. We therefore believe that for the younger and fitter men, the excellent D90/BED dosimetry outcomes supplemented by short-term ADT, do result in the best clinical outcomes in the long run. We do not support indiscriminate use of ADT especially in the elderly and frailer men.

Contrariwise we suggest that the addition of short-term ADT to brachy-monotherapy to men in the Gleason 6 intermediate risk group, does not result in better bPFS data, may not be sufficient and therefore not justified. Analyzing our excellent outcome with combining brachytherapy, EBRT and short term ADT for intermediate Gleason score 7 disease, suggest the potential beneficial role of combining both radiation methods with ADT in this group of men. In the absence of a group of men receiving combination of radiation but with no ADT we cannot be more decisive. Stone and Stock [13] in their earlier experience favored the addition of 9-month ADT to the combination of both radiation modalities. Somewhat later, they specifically looked at D'Amico intermediate risk group patients treated with combination brachytherapy and EBRT and their 8 years biochemical failure free rates were identical between those treated with and without ADT. Specifically in the Gleason 7 intermediate risk group patients and among those with PSA 10–20 ng/ml the ADT therapy had no significant impact on biochemical failure-free rates. They attributed this observation to the high BED achieved by the authors and question the routine use of ADT in the intermediate risk set-up. A more recent updated report on 15-yr cause-specific and all-cause mortality rates still questions the addition of ADT for this intermediate-risk group of patients [32]. Among different risk groups and brachytherapy as monotherapy or in combination with EBRT, hormone therapy did not positively impact cause specific survival and did not reduce all-cause mortality. This was seen for all age groups treated. Castle et al. [33] in a favorable subset of intermediate risk group, mainly Gleason score 6 men, have reported almost identical 5-yr bPFS with or without ADT (94% vs 95%). In a multivariate analysis of their group, again ADT was not predictive of bPFS. We do believe that prospective randomized trials are needed to further elucidate the role of ADT in this particular group of patients, weighing side-effects vs. survival and perhaps reporting not only bPFS rates but overall survival.

Ohashi et al. [3] evaluated biopsy positive core rates and in a cox multivariate regression analysis found the core positive rate was noncontributory and only D90 doses were predictive of failure. The same group [3] also evaluated percent positive core rates in high-risk men treated with combination brachytherapy and EBRT. In this particular group in a multivariate analysis the 5 years biochemical failure-free rates were influenced by core positivity (>50% vs. <50%) as well as the number of high risk factors. At 60-month median follow-up, this difference although significant at 0.03, was not considered by the authors to be of high clinical relevance concluding that with the high radiation doses achieved even without ADT there was still a high probability of achieving good 5-yr biochemical failure-free rates. Merrick et al. [34] examined their group of 262 men treated with low dose radiotherapy (LDR) as monotherapy in all 3 risk categories, of which 215 had detailed information on percentage of positive cores. A significant trend was found for biochemical failure with increasing positive cores in the overall population. This could not be demonstrated when each risk group was looked at separately. They too attributed this lack of significance to the escalated radiation doses achieved with brachytherapy and to better treated periprostatic region compared to radical prostatectomy. Our current report could demonstrate only borderline significance for percent core positivity. This may be internally correlated with PSAD, since PSAD may be used as a proxy for disease volume and may indeed perhaps be a better variable to look at that minimizes the effect of the randomized biopsies upon which the percent core variable is based upon. The much more homogenous and higher D90 dosimetry outcomes may be part of this observed difference between various reports.

The limitations of our study, some of it already mentioned, are the retrospective nature of the report, although follow-up was prospectively planned and gathered. Our inability to further differentiate Gleason into 4+3 and 3+4, a difference we now know is of high clinical importance and the lack of randomization between those given ADT and those who were not is also an acknowledged limitation.

Our large number of patients, and the small number of patients lost to follow-up as well as a rather long follow-up period we believe still adds to our current knowledge. However, this retrospective study should be regarded as a hypothesis generator only and necessitates prospective studies in GS 7 intermediate risk patients in order to clarify the role of either the addition of EBRT and/or ADT, some of which are luckily currently underway.

## Conclusions

Brachytherapy provides excellent long-term bPFS rates in low and intermediate risk disease. Our retrospective analysis, a hypothesis generating study suggests that combination of brachytherapy with EBRT yields favorable outcomes in GS 7 intermediate risk patients and short-term ADT has a positive effect on outcomes in low risk patients.

## Supporting information

S1 Fig CONSORT flow diagram of prostate cancer study patients. GS, Gleason score; PSA, prostate-specific antigen; Brachy, brachytherapy; ADT, androgen-deprivation therapy; EBRT, external beam radiotherapy.
(PDF)

## Abbreviations

bPFS: biochemical progression-free survival
ADT: androgen deprivation therapy
EBRT: external beam radiotherapy
PSA: prostate-specific antigen
PSAD: PSA PSA density
GS: Gleason score
BED: Biological effective dose
SD: standard deviation
IQR: interquartile range
PPC: percent of positive cores
