# Supplementary material for: Long-term biochemical progression-free survival following brachytherapy for prostate cancer: Further insight into the role of short-term androgen deprivation and intermediate risk group subclassification
Source: PLoS One. 2019 Apr 19;14(4):e0215582. doi: 10.1371/journal.pone.0215582 (PMC6474628; doi:10.1371/journal.pone.0215582)
Supplement: S1 Fig — GS, Gleason score; PSA, prostate-specific antigen; Brachy, brachytherapy; ADT, androgen-deprivation therapy; EBRT, external beam radiotherapy. (PDF) [file pone.0215582.s001.pdf]

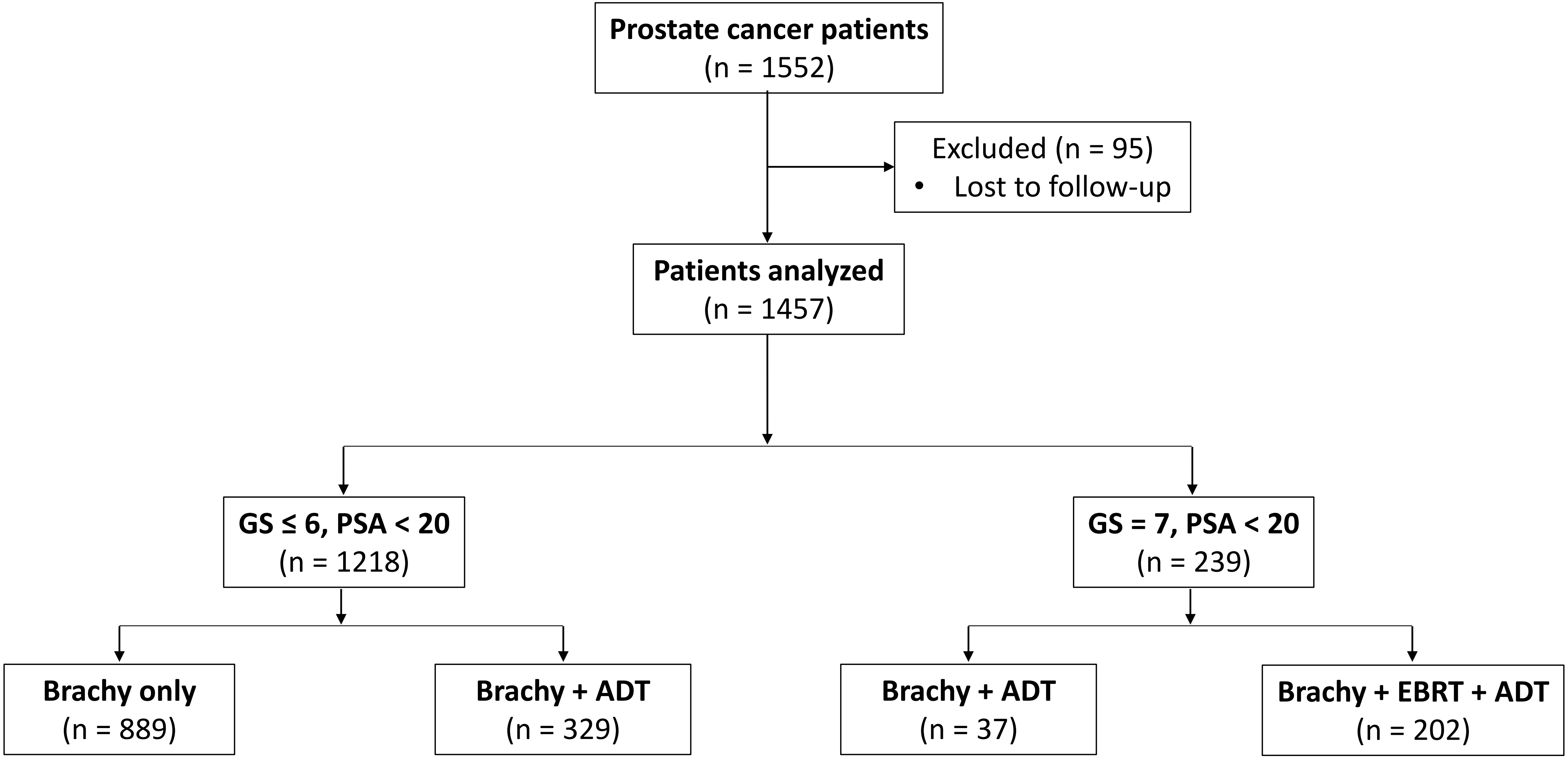

Prostate cancer patients
(n = 1552)
Excluded (n = 95)
• Lost to follow-up
Patients analyzed
(n = 1457)
GS ≤ 6, PSA < 20
(n = 1218)
GS = 7, PSA < 20
(n = 239)
Brachy only
(n = 889)
Brachy + ADT
(n = 329)
Brachy + ADT
(n = 37)
Brachy + EBRT + ADT
(n = 202)
